# Clinical and Radiological Outcomes of Total Knee Arthroplasty Performed with Midvastus and Medial Parapatellar Approaches in Obese Patients

**DOI:** 10.1155/2021/5512930

**Published:** 2021-05-10

**Authors:** Olcay Guler, Gürkan Gümüşsuyu, Hakan Sofu, Hüseyin Bahadır Gökçen

**Affiliations:** ^1^Department of Orthopedics and Traumatology, Medical Faculty, Altınbaş University, Istanbul, Turkey; ^2^Department of Orthopedics and Traumatology, Medical Faculty, Istinye University, Istanbul, Turkey

## Abstract

**Background:**

The use of total knee arthroplasty (TKA) for primary osteoarthritis of the knee has remarkably increased recently. We aimed to compare the clinical and radiological outcomes of TKA in obese patients (>30 kg/m^2^) operated with midvastus (MV) or medial parapatellar (MPP) approaches.

**Methods:**

This retrospective study was performed using data derived from 80 patients (70 women; 10 men) with an average age of 66.17 ± 5.42 (range: 54 to 77). Patients were allocated into 2 groups as for the type of approach conducted during TKA: group I (*n* = 41) underwent TKA by MV approach, while the MMP technique was used in group II (*n* = 39).

**Results:**

Demographic, clinical, and radiological parameters included age, side of involvement, sex, BMI, diameters of thigh and calf, length of incision, duration of operation, amount of bleeding and transfusion, duration of hospitalization and follow-up, complications, and range of motion, as well as Knee Society Score (KSS) and Knee Society Function Score (KSFS). Patients with a higher BMI (≥35 kg/m^2^) experienced more profound bleeding and needed more transfusion of erythrocyte suspension. The range of motion was more favorable in groups with BMI <35 kg/m^2^. The functional outcomes as reflected in KSS and KSFS were much better in patients with BMI <35 kg/m^2^.

**Conclusions:**

Our data indicated that obesity can adversely influence the clinical and radiological outcomes after TKA performed by both MV and MPP approaches. A careful analysis of patient characteristics and selection of appropriate operative procedures is critical. Further randomized, controlled trials on larger series must be designed to elucidate the relationship between obesity and therapeutic outcomes after TKA with different approaches.

## 1. Introduction

Total knee arthroplasty (TKA) is considered the best treatment method for end-stage osteoarthritis [1]. It is performed to correct deformity, relieve pain, and restore joint function in arthritic knees [2]. The medial parapatellar (MPP) approach is accepted as a simple and standard approach for TKA, which provides adequate exposure of the joint [2, 3]. Nevertheless, this method brings about risks such as the split of the quadriceps tendon, reduction of the patellar blood supply, and diminution of extensor strength. These drawbacks may lead to complications such as patellar fracture and patellofemoral instability [2, 3]. On the other hand, the midvastus (MV) approach was developed as an option to MPP, and it allows the preservation of the quadriceps tendon intact. The MV offers advantages such as better quadriceps control, earlier functional outcomes, less pain, and better patellar tracking. On the other hand, MV may be associated with problems like neurovascular injury and insufficient exposure. There is still controversy on the preference of MPP and MV routes for TKA [2–4].

Obesity is a frequent condition that increases the need for TKA. There is debate on the risks, benefits, and complications of TKA in obese patients. An accurate understanding of the medical and surgical complications associated with TKA in the obese patients will enhance research efforts and improve outcomes [5].

The purpose of the present study was to compare the clinical and radiological outcomes of TKA with MV and MPP approaches in obese patients.

## 2. Patients and Methods

### 2.1. Study Design

This retrospective study was performed in the orthopedics and traumatology department of our tertiary care center. The approval of the local institutional review board was obtained before the study. Data was gathered from the medical files of a total of 80 patients (70 women; 10 men) who underwent TKA for primary osteoarthritis of the knee using MV or MPP approaches. The average age of our series was 66.17 ± 5.42 (range: 54 to 77). Patients had undergone surgery between January 2014 and January 2019. Patients were allocated into 2 groups as for the type of approach conducted during TKA: group I (*n* = 41) underwent TKA by MV approach while the MMP technique was used in group II (*n* = 39).

Patients aged 18 to 80 years with a body mass index (BMI) >30 kg/m^2^ were included. Patients with BMI <35 were termed group Ia and group IIa, whereas patients with BMI ≥35 were named as group Ib and group IIb. Exclusion criteria were collagen vascular diseases, history of high tibial osteotomy, flexion angle <80°, valgus and varus deformity >30°, previous history of infection of knee joint, neurological deficit such as diabetic neuropathy, previous history of fracture or surgery of the femur and tibia, and osteoarthritis of the ipsilateral or contralateral hip joint.

### 2.2. Surgical Procedure

Spinal or epidural anesthesia was routinely used in all patients. An anterior cruciate ligament preserving total knee replacement prosthesis (*Genesis 2, Smith & Nephew, Memphis, TN, USA*) was utilized. Tourniquet was not used in any of our patients.

**Figure 1:**
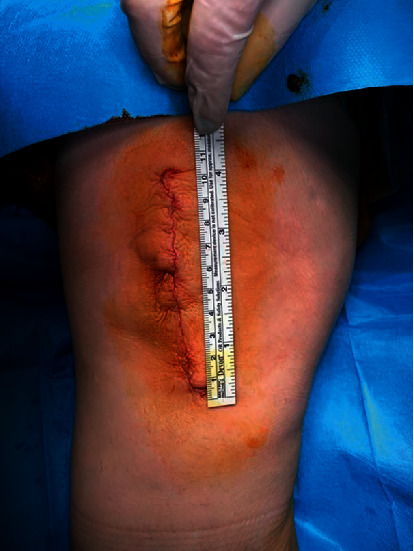
The measurement of incision length at extension of the knee.

In group I, a skin incision passing through 1/3 medial of the patella, which starts from 1 to 2 cm proximal of the patellar upper pole, extends toward 2 cm distal to knee joint line, and ends at the medial border of tibial tuberosity, was performed (Figure 1).

The exposure to the patellar retinaculum was provided longitudinally; starting 1 cm medial to the patella, the capsular incision was extended from 2 cm proximal to the upper pole of the patella. The incision passed 2–4 cm inside the vastus medialis oblique (VMO) muscle bundles. Patella was dislocated laterally without eversion. In group I, specially designed small instruments were used to carry out distal femoral incisions for allowing 6° of valgus in the coronal plane and providing 90° exposure to the anterior-posterior distal femur in the sagittal plane. The femoral rotation was adjusted using posterior condylar and epicondylar axes. Tibial incisions were performed 90° on the coronal plane and 7° on the posterior tibial slope on the sagittal plane. An extramedullary guide was used on the tibia and an intramedullary guide was used on the femur. Lateral release and patellar resurfacing were not performed in any patients. No thumb test was performed to assess patellar alignment.

In group II, a straight incision that passes through the midpatellar line was performed while the knee was maintained at flexion. After the patellar retinaculum was incised longitudinally through the medial patella, a capsular incision was extended proximally to the union of rectus femoris and VMO muscles and 2 cm below tibial tuberosity distally. Patella was dislocated laterally without eversion and the knee joint was maintained at flexion. Using specially designed instruments, femoral incisions were performed with an intramedullary guide with a 90° angle in the sagittal plane on the anterior and posterior distal femur. The femoral rotation was arranged using posterior condylar and epicondylar axes. After exposure to the tibia, tibial incisions were carried out using an extramedullary guide. Lateral release and patellar resurfacing were not performed in any patients. Patella tracking was evaluated using a no thumb test.

All patients received tranexamic acid at a dose of 1 g via the intravenous route (*Transamine*®*, Mefar Pharmaceuticals, Istanbul, Turkey*) before and after the operation. Moreover, periarticular tranexamic acid injections were performed perioperatively. All patients had low molecular weight heparin (*Clexane*®*, Sanofi, Istanbul, Turkey*) for 21 days for prophylaxis against deep venous thrombosis.

### 2.3. Postoperative Care and Follow-Up

The drain was removed on the 1st day postoperatively and the patient was mobilized with crutch Canadian canes. In addition to the initiation of passive joint motion with a continuous passive motion (CPM) device, isometric quadriceps and active knee flexion and extension exercises were started on the first postoperative day.

For the relief of postoperative pain, narcotic analgesics were administered with patient-controlled analgesia (PCA) on the first postoperative day. On the following, intravenous and oral analgesics were administered. Prophylactic antibiotics were given for 24 hours. Patients were controlled on 15 days, 6 weeks, 3 months, 6 months, 12 months, and annually every year after the procedure. Evaluation of the axis using low extremity roentgenography and calculation of mechanical femorotibial angle were carried out before surgery and on the 3rd month postoperatively.

All radiological measurements were performed on Picture Archiving and Communication System (PACS). These steps were carried out by 2 experienced surgeons who were not involved in the surgical team and who were blinded to patient data. These surgeons conducted measurements twice with 1-month intervals.

Superficial infections were treated with oral antibiotics. In patients with a patellar tendon injury, knee pads were used for 4 weeks since patients could perform active straight leg raise.

### 2.4. Outcome Measures

The main outcome measures were age, side of involvement, sex, BMI, diameters of thigh and calf, length of incision, duration of operation, amount of bleeding and transfusion, duration of hospitalization and follow-up, complications, time of straight leg raise (days), pre- and postoperative mechanical femorotibial angle measurements in terms of coronal and sagittal femoral and tibial slopes, range of motion, Knee Society Score (KSS), and Knee Society Function Score (KSFS) [6, 7].

**Figure 2:**
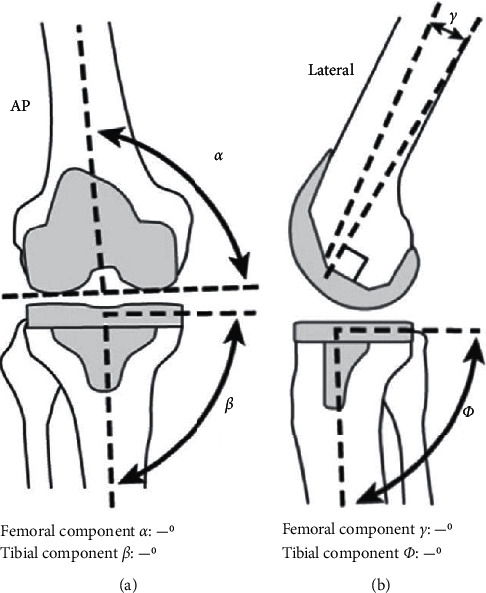
Roentgenographic evaluation parameters after total knee arthroplasty (TKA) (Ewald FC).

The parameters of the radiographic evaluation system were coronal femur alpha (medial distal femoral angle: MDFA), coronal tibia (beta), sagittal femur (lambda), and sagittal tibia (gamma) (Figure 2) [8, 9].

The range of motion was assessed in terms of flexion and extension and the clinical and functional outcomes after TKA as for KSS and KSFS were evaluated preoperatively, on 2 weeks, 6 weeks, 3 months, 6 months, 12 months, and on the final visit.

**Figure 3:**
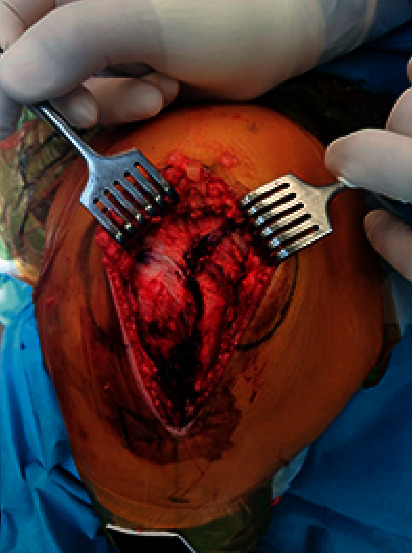
Preoperative image demonstrating midvastus approach.

The diameters of the thigh and calf were measured in the midthigh and midcalf, respectively. The duration of surgery was described as the interval between the onset of incision and the placement of the final suture (Figure 3). The amount of intraoperative bleeding (mL) was calculated using the blood collected in suction (the volume of irrigation fluid was excluded), the weight of gauze, and blood collected in the drain. The length of the incision was measured at extension.

### 2.5. Statistical Analysis

Our data were analyzed with Statistical Package for Social Sciences version 21.0 for Windows software (*SPSS Inc., Chicago, Illinois, USA*). Descriptive data were presented as counts and percentages. Quantitative data were expressed as mean, standard deviation, median, minimum, and maximum. The normal distribution of variables was tested using the Kolmogorov–Smirnov test. For continuous variables, the student's *t*-test was employed as a parametric test, whereas Mann–Whitney *U* and Kruskal–Wallis tests were used as nonparametric tests. The Pearson chi-square test was utilized for the evaluation of categorical variables. A *p* value less than 0.05 was considered statistically significant.

## 3. Results

A comparative overview of demographic variables under investigation is presented in Table 1. Our population consisted of 80 patients (70 women; 10 men) with an average age of 66.17 ± 5.42 (range: 54 to 77). Groups I and II were further classified into 2 groups as for BMI. Patients with BMI <35 were termed group Ia and group IIa, whereas patients with BMI ≥35 were named as group Ib and group IIb.

The clinical and perioperative variables in 2 groups are demonstrated in Table 2. Radiographic parameters before and after TKA and the range of motion and functional scores before and after surgery are shown in Tables 3 and 4, respectively.

These 4 groups revealed similar results in terms of age (*p*=0.171), sex distribution (*p*=0.293), side of involvement (*p*=0.674), duration of follow-up (*p*=0.634), preoperative mechanical femorotibial angle (*p*=0.383), preoperative flexion (*p*=0.779), extension on postoperative 2nd week (*p*=0.166), and diameters of thigh (*p*=0.573) and calf (*p*=0.639) (Tables 1–4).

Remarkably, the amount of bleeding was more profound (*p* < 0.001), BMI was higher (*p* < 0.001), postoperative mechanical femorotibial angle results were increased (*p*=0.001), and the preoperative extension range was more extensive (*p*=0.029) in groups Ib and IIb. The length of the incision was longer in group Ib (*p*=0.001). The amount of erythrocyte suspension transfusion was higher in groups Ib and IIb (*p* < 0.001) (Table 2).

Flexion on the 2nd week, 6th week, and 3rd month postoperatively was higher in group IIa (*p* < 0.001, for all). Flexion on the 3rd month postoperatively was higher in group IIa (*p* < 0.001) (Table 4).

Extension on the 3rd month postoperatively was higher in group IIb (*p*=0.012). Flexion on the 6th and 12th months postoperatively was higher in groups Ia and IIa (*p*=0.001) (Table 4).

Extension on 6th and 12th months postoperatively was higher in groups Ib and IIb (*p*=0.012 and *p*=0.028, respectively). Flexion on the final control visit was remarkably lower in groups Ib and IIb (*p*=0.001). On the other hand, the extension on the final control visit was higher in groups Ib and IIb (*p*=0.048) (Table 4).

Radiographic parameters such as coronal femoral alpha, coronal tibia beta, sagittal femoral lambda, and sagittal tibial gamma displayed remarkable differences in both measurements performed by 2 independent observers.

The KSS and KSFS preoperatively, on 6 weeks, 3 months, 6 months, 12 months, and on the final visit postoperatively were remarkably higher in groups Ia and IIa (Table 4).

In group I, a skin blister was detected in 1 patient and a superficial infection treated successfully with oral antibiotics was diagnosed in 1 patient. In group II, skin blisters were observed in 2 patients and superficial infection was identified in 1 patient.

## 4. Discussion

TKA provides satisfactory and reproducible results, and the MPP approach has been used as the standard technique before minimally invasive approaches were devised. Despite the fact that MP is a successful method, the recovery period can be prolonged and a rehabilitation period of up to 1 year can be necessary before prompt functional recovery. The midvastus method is one of the minimally invasive techniques and it may allow faster functional recovery, shorter duration of hospitalization, and more favorable ROM. However, the debate on the advantages and disadvantages of minimally invasive techniques is not resolved [10].

Our findings yielded that bleeding and the need for transfusion of erythrocyte suspension were more frequent in obese patients who underwent TKA. The functional outcomes as reflected in KSS and KSFS were more favorable in nonobese patients. Similarly, nonobese patients displayed better outcomes in terms of the range of motion and radiography compared to that of obese patients.

Efforts have been spent to determine the optimal surgical modality with satisfactory safety and effectivity in TKA [11]. The prevalence of obesity was found to be increased in patients undergoing primary, revision, and infected TKA. The obesity epidemic seems to be linked with the increased likelihood of revision and infection after surgery. Since the rates of obesity may further increase, it can be estimated that the burden of revision and infection related to obesity may be more obvious [12].

Li et al. suggested that MV offers a better range of motion and more effective pain control than MPP in the immediate postoperative period after TKA [2]. Postoperative knee function is the most important criterion for the assessment of the effectiveness of the procedure. The MV approach can yield more favorable outcomes in the early recovery period compared to the MPP method. On the other hand, restoration of appropriate patella tracking is crucial for postoperative recovery. The midvastus approach can provide more acceptable patella tracking due to the preservation of patellar tendons which keep the patella in its original position. No remarkable differences were noted between the 2 approaches in terms of alignments of the knee, tibial and femoral components, posterior slope, and bone-patellar angle [2].

Bourke et al. proposed that trials should have an adequate sample size, preoperative baseline assessment of pain and function, physical measurements of the knee, intraoperative data including operative time and blood loss, follow-up for at least 6 to 12 months, and length of hospitalization [13].

It must be remembered that the duration of hospitalization after TKA can be affected by many factors including the overall healthcare system, demographic features, social and environmental factors, and perioperative management [6]. We suggest that loss of weight may lead to improved functional outcomes as reflected in KSS and KSFS. The KSS, which is an outcome measure related to the range of motion, was reported as the primary concern by the patients [11]. Our data imply that the musculoskeletal system and joint procedures such as TKA are directly affected by weight gain and an increase in BMI.

Xu et al. reported that the MV approach was more effective in an improvement of VAS and ROM in short term. However, it was associated with prolonged operative time. These aspects must be remembered during tailoring the treatment strategy for primary osteoarthritis of the knee [11].

Obesity is supposed to make surgery more challenging in TKA, and it has been termed a potential contraindication to the subvastus approach [14, 15]. We compared the MV and MPP approaches and our preliminary data is insufficient to address obesity as a contraindication for these 2 methods. Notably, we detected that the amount of erythrocyte suspension transfusion was higher in patients with BMI of ≥35. These findings are important since it reminds that risks of obesity will be further amplified by risks associated with increased likelihood for transfusion.

The single-use instruments offer advantages like the reduction of costs, timely turnover of operating rooms, and decreased rate of infection. No difference has been observed between single-use and conventional instruments as for clinical outcomes and radiographic parameters. These data imply that SUI can be an alternative to conventional instruments; however, further trials are warranted to compare the clinical outcomes [16].

To the best of our knowledge, this is the first study focusing on the comparison of 2 surgical methods in obese patients scheduled for TKA. The main limitations of the present study include retrospective design, heterogeneity of gender distribution, relatively small sample size, and data restricted to the experience of a single center. The main strengths of this trial were simultaneous evaluation of clinical, radiological, and perioperative parameters. In other words, both physical and functional measures were included in the evaluation of therapeutic outcomes. These points should be considered during the interpretation of our results.

## 5. Conclusion

Obesity is a growing health problem, and it may directly affect the surgical outcomes in TKA. This procedure can be carried out using MV and MPP approaches. We noted that obesity could influence the clinical and radiological outcomes after TKA performed by using MV and MPP approaches. A careful analysis of patient characteristics and selection of operative procedure is critical, and BMI is an important parameter to be considered during the establishment of the treatment plan. Further multicentric, randomized, controlled trials on larger series must be designed to verify our findings and to understand the association between obesity and therapeutic outcomes after TKA.

## Figures and Tables

**Table 1:** A comparative overview of demographic variables under investigation.

Variable	Group				*p* value
	Ia	Ib	IIa	IIb	
Age (years)	63.67 ± 5.95	68.53 ± 5.18	66.57 ± 5.07	66.00 ± 5.69	0.171
Sex (F/M)	22/2	14/3	26/2	8/3	0.293
Body mass index (kg/m^2^)	32.79 ± 1.47	36.65 ± 1.00	33.18 ± 1.61	36.91 ± 0.83	**<0.001**
Side of involvement (R/L)	15/9	13/4	17/11	8/3	0.674

**Table 2:** A comparative overview of clinical and perioperative variables under investigation.

Variable	Group				*p* value
	Ia	Ib	IIa	IIb
Bleeding (mL)	31.35 ± 12.62	54.80 ± 17.04	28.73 ± 9.44	66.23 ± 20.95	**<0.001**
Follow-up (months)	38.58 ± 10.96	35.12 ± 14.20	36.21 ± 13.52	39.09 ± 11.38	0.634
Diameter of thigh (cm)	52.92 ± 3.59	62.41 ± 4.46	52.64 ± 5.89	63.82 ± 4.28	0.573
Diameter of calf (cm)	34.83 ± 4.00	44.59 ± 3.68	37.04 ± 5.37	43.82 ± 3.63	0.639
ES transfusion (units)	1.25 ± 0.61	2.06 ± 0.56	1.25 ± 0.70	2.36 ± 0.51	**<0.001**

**Table 3:** Radiographic parameters before and after TKA.

Preoperative MFTA (°)		190.63 ± 3.63	190.29 ± 2.73	190.50 ± 3.25	193.27 ± 4.82	0.383
Postoperative MFTA (°)		180.54 ± 1.38	181.47 ± 1.62	180.54 ± 1.17	182.45 ± 1.57	**0.001**
Coronal femur (alpha)^*∗*^	I	122.50 ± 5.32	120.59 ± 3.48	124.46 ± 4.97	117.73 ± 5.18	**0.002**
Coronal tibia (beta)^*∗*^	I	89.42 ± 1.18	88.88 ± 1.17	89.64 ± 1.16	88.45 ± 0.93	**0.008**
Sagittal femur (lambda)^*∗*^	I	0.79 ± 1.02	1.59 ± 1.23	1.11 ± 1.42	2.55 ± 1.37	**0.002**
Sagittal tibia (gamma)^*∗*^	I	84.88 ± 0.68	84.65 ± 0.79	85.21 ± 0.88	84.36 ± 1.63	**0.018**
Coronal femur (alpha)^*∗*^	II	64.58 ± 3.27	62.06 ± 2.54	72.86 ± 6.00	67.27 ± 4.67	**<0.001**
Coronal tibia (beta)^*∗*^	II	88.71 ± 1.33	89.29 ± 1.16	89.39 ± 1.29	89.82 ± 0.60	**0.045**
Sagittal femur (lambda)^*∗*^	II	1.54 ± 1.06	0.88 ± 1.22	1.21 ± 1.26	2.36 ± 0.92	**0.021**
Sagittal tibia (gamma)	II	85.21 ± 0.98	85.06 ± 1.03	84.75 ± 0.97	84.91 ± 0.70	0.302
Coronal femur (alpha)^§^	I	95.25 ± 0.85	94.88 ± 0.86	95.07 ± 0.72	95.00 ± 0.78	0.378
Coronal tibia (beta)^§^	I	95.08 ± 0.83	94.94 ± 0.80	89.54 ± 0.96	89.09 ± 1.22	**<0.001**
Sagittal femur (lambda)^§^	I	1.42 ± 1.28	1.41 ± 0.94	1.75 ± 1.04	1.18 ± 1.25	**<0.001**
Sagittal tibia (gamma)^§^	I	89.54 ± 1.41	89.29 ± 1.05	85.46 ± 0.79	85.55 ± 0.79	**<0.001**
Coronal femur (alpha)^§^	II	85.04 ± 0.95	84.76 ± 1.15	95.07 ± 0.72	95.27 ± 1.10	**<0.001**
Coronal tibia (beta)^§^	II	88.78 ± 1.27	89.12 ± 1.07	89.54 ± 1.00	89.55 ± 1.21	**<0.001**
Sagittal femur (lambda)^§^	II	1.38 ± 0.77	1.24 ± 1.03	1.64 ± 1.13	1.55 ± 0.93	**<0.001**
Sagittal tibia (gamma)^§^	II	85.25 ± 0.94	85.00 ± 1.12	84.93 ± 1.02	85.27 ± 0.90	0.516

**Table 4:** Range of motion and functional scores before and after surgery.

Preoperative flexion	94.38 ± 6.81	96.47 ± 6.56	96.61 ± 6.81	93.18 ± 7.17	0.779
Preoperative extension	5.00 ± 3.90	8.24 ± 2.46	5.71 ± 4.24	8.18 ± 4.62	**0.029**
Flexion (postoperative 2 weeks)	82.92 ± 5.88	80.00 ± 5.86	91.43 ± 8.70	83.18 ± 6.03	**<0.001**
Extension (postoperative 2 weeks)	3.33 ± 3.18	5.00 ± 3.54	3.21 ± 3.39	5.45 ± 4.16	0.166
Flexion (postoperative 6 weeks)	105.21 ± 7.44	100.00 ± 5.86	113.39 ± 7.82	104.55 ± 10.60	**<0.001**
Extension (postoperative 6 weeks)	0.63 ± 1.69	1.47 ± 2.35	0.36 ± 1.89	2.73 ± 3.44	**0.037**
Flexion (postoperative 3 months)	118.75 ± 5.16	117.06 ± 3.98	123.21 ± 4.76	116.82 ± 5.13	**<0.001**
Extension (postoperative 3 months)	0.42 ± 1.41	1.18 ± 2.19	0.18 ± 1.66	2.73 ± 3.44	**0.012**
Flexion (postoperative 6 months)	120.53 ± 5.02	118.97 ± 3.60	123.26 ± 4.50	117.42 ± 5.10	**0.001**
Extension (postoperative 6 months)	0.40 ± 0.92	1.10 ± 2.05	0.16 ± 1.40	2.40 ± 2.70	**0.011**
Flexion (postoperative 12 months)	122.92 ± 5.30	120.88 ± 3.18	124.64 ± 4.45	118.18 ± 5.14	**0.001**
Extension (postoperative 12 months)	0.40 ± 1.37	1.14 ± 2.10	0.34 ± 1.67	2.73 ± 3.12	**0.048**
Preoperative KSS	40.88 ± 6.95	38.88 ± 5.48	42.75 ± 6.94	35.55 ± 3.42	**0.014**
Preoperative KSFS	37.92 ± 5.70	37.06 ± 5.32	39.64 ± 5.08	32.73 ± 5.18	**0.010**
KSS (postoperative 6 weeks)	83.00 ± 3.50	80.00 ± 2.12	83.61 ± 3.76	78.55 ± 4.89	**<0.001**
KSFS (postoperative 6 weeks)	81.67 ± 4.34	78.82 ± 3.76	82.50 ± 3.72	76.82 ± 4.62	**0.001**
KSS (postoperative 3 months)	86.33 ± 2.87	83.35 ± 1.77	87.86 ± 2.80	83.36 ± 2.58	**<0.001**
KSFS (postoperative 3 months)	86.88 ± 3.85	83.24 ± 3.03	86.79 ± 4.13	82.27 ± 5.18	**0.002**
KSS (postoperative 6 months)	87.29 ± 2.48	84.41 ± 1.80	88.32 ± 2.37	84.64 ± 3.26	**<0.001**
KSFS (postoperative 6 months)	87.29 ± 3.60	85.29 ± 2.78	87.32 ± 4.19	82.73 ± 5.64	**0.015**
KSS (postoperative 12 months)	87.46 ± 2.38	84.82 ± 1.42	88.71 ± 2.36	85.73 ± 2.00	**<0.001**
KSFS (postoperative 12 months)	86.42 ± 2.66	85.53 ± 2.34	87.62 ± 4.01	81.73 ± 4.28	**0.002**
KSS (final control)	87.63 ± 2.34	84.88 ± 1.45	88.86 ± 2.42	85.73 ± 2.00	**<0.001**
KSFS (final control)	87.75 ± 2.91	85.59 ± 2.42	87.68 ± 4.19	82.73 ± 5.64	**0.003**

## Data Availability

The data used to support the findings of this study are available from the authors upon request.

## Abbreviations

F: Female
M: Male
R: Right
L: Left
ES: Erythrocyte suspension
MFTA: Mechanical femorotibial angle
I: Initial measurement
II: Second measurement
^*∗*^: First independent researcher
^§^: Second independent researcher
KSS: Knee Society Score
KSFS: Knee Society Function Score.
